# Creating Incentives to Improve Population Health

**Published:** 2010-08-15

**Authors:** Steven Lewis

**Affiliations:** Access Consulting Ltd. Mr Lewis is also affiliated with the University of Calgary, Calgary, Alberta, Canada, and Simon Fraser University, Burnaby, British Columbia, Canada

## Introduction

How do communities improve the health of their populations? For the past century, we have not been required to think deeply about the question because health status steadily improved. Life expectancy increased by 30 years in prosperous countries between 1900 and 2000. But now the question is emerging as one of the most important we face. The rate of "natural" improvement in health status appears to be slowing, and decline is not unthinkable if the sharp rise in the prevalence of chronic conditions such as obesity and type 2 diabetes continues unabated. Research identifying the nonmedical determinants of health has flourished in recent decades. The correlations are well understood, but the causes of health disparities and the extent to which they can be mitigated remain debatable. How do societies come to take population health improvement seriously? One potential pathway is incentives.

## Some Sobering Realities

Improving health care is hard; improving population health is even harder, as the articles in this issue of *Preventing Chronic Disease* discuss. Decades of analysis and experimentation have confirmed the following:

1. Targets can be useful but also distracting and unintentionally destructive to the population health agenda (1).

2. Little evidence supports the proposition that population health can be improved with resources freed up by making health care more effective and efficient (2).

3. Pay for performance, so attractive in theory, is fraught with difficulties in practice, among them methodologic problems and moral hazard. As typically understood and deployed, the concept may be particularly inimical to a population health agenda (3).

4. Health status variability is inevitable, but even people who are born with identical health status will have diverse outcomes over the life course because of circumstances and choices. Moreover, establishing causation is elusive because of the complexity of factors that affect the health of both people and communities and the danger of being seduced by ecological fallacies. Some also argue, more controversially, that if health is to improve, we must give up other social goods; the laws of scarcity apply, and there is no positive-sum scenario (4).

5. Experiences in other sectors reveal the mixed and sometimes unforeseeable effect of incentives. In education they have worked in some instances but have also resulted in perverse behaviors (eg, gaming, adverse selection). Creating effective incentives for particular circumstances is challenging, ensuring that the incentives evolve as circumstances change even more so. Many jurisdictions have abandoned merit pay schemes for teachers, and the effect of teacher certification programs appears to have been modest. In health care, a combination of high-quality comparative evidence and incentives is insufficient to achieve the desired practices and outcomes (5). Producing effective and durable reward systems is difficult in health care, and more difficult still in population health (6).

6. Some policies could plausibly improve population health if applied more vigorously. Some effective programs fail not because of their inherent logic and structure but because of low uptake. For example, 67% of people who are eligible for food stamps are not enrolled in the program (7). Performance measures and rankings can create awareness and a growing sense of responsibility for addressing population health needs and inequalities. However, it is important to sort out whether community health status is a dependent variable (based on how well systems and programs perform), an independent variable (based on need), or both (8).

## What Is to Be Done?

Notwithstanding these methodologic challenges, a thread of optimism runs through the incentives articles. Some authors propose that as the evidence gets stronger and more compelling, policy makers will eventually do the right thing. Knowledge about population health inequalities is deep and diverse, but links between policies or incentives and population health outcomes are not well documented. The implicit argument is that a critical mass of demonstration projects, evaluations, and case studies will ultimately have the intended effect on politics and society, and change will occur.

Unfortunately, there is reason for skepticism. If we conceive of population health improvement in terms of reduced disparities, benefits must increasingly concentrate on populations of low socioeconomic status. Experience suggests that narrowing disparities is extraordinarily difficult. We are limited in our understanding of the factors that produce better population health, but evidence suggests that societies with less inequality are healthier (9). The problem is not that we have no clue about how to improve population health or that people oppose improving the health of disadvantaged populations in principle. The problem is that there is no strong political commitment to the pursuit of these aims, no political liability inherent in not achieving them, and no consensus that this goal should be pursued more ardently than other goals (that may actually exacerbate inequalities). The sciences of epidemiology and biostatistics explain the nature, extent, and consequences of population health inequalities, but we must look to the political arts to understand why they are so hard to mitigate.

At the heart of the political dilemma is the reality that population health improvement is but one of many competing values. Individuals and communities steeply discount future health benefits, and population health improvement is a long, winding process whose ultimate benefits may take decades to quantify. A similarly steep discount applies to saving or improving anonymous, aggregate lives compared with individual lives with names and faces. A third factor that steepens the discount rate is that society values health gains attributable to health care interventions more than those achieved through social and economic policies and interventions. In a political context, how health is improved matters as much as whether it is improved — or so it would seem, judging from our enormous investment in health care that delivers virtually zero at the margins, and from the beggaring of investment in nonmedical improvement strategies.

A large public has been persuaded of the value of increasingly specialized and sophisticated health care and health technology, despite the clear absence of effect on health status. This symbolic and empirical devotion to health care is a formidable challenge to a population health agenda. In Canada, we could eliminate poverty (as defined by Statistics Canada's low-income cutoff) for $25 billion annually (10) — about 20% of publicly financed health care spending. No one is in favor of poverty, but political sentiment does not favor reallocating any part of health care spending to its elimination.

If this analysis is plausible, it follows that generating broader political commitment to population health improvement has to appeal to democratically shared and expressed values that can be converted into a feasible political agenda. But should this case be cast in terms of population health and disparities reduction as the goal of policy, or as the happy effect of the pursuit of other objectives such as economic productivity, reduction in crime and social problems, international competitiveness, and general well-being? The Canadian Index of Wellbeing (11) has been developed to introduce concepts and measures of societal performance that are more meaningful and comprehensive than economically focused measures such as gross domestic product.

We should not overlook the potential contribution of accountants. The costs of disparities are enormous (eg, poorly educated and therefore unproductive citizens; crime, law enforcement, and incarceration; excessive use of health care that may be ineffective; safety surveillance systems). If voters, particularly the middle class, can be persuaded to endorse policies that enhance population health, governments may respond accordingly.

These reflections may lead to a sense of hopelessness and even nihilism, but we should not confuse a political dilemma with categorical impossibility. Suppose there were literally guns at the temples of senior policy makers, set to go off in 5 years in the absence of emerging evidence of population health improvement and in 10 years in the absence of concrete improvement. I, for one, have no doubt they would survive. If nearly $800 billion can be authorized in months to stimulate the economy (12), imagine the effect of a small fraction of that amount spent on universal child care, Head Start, micro-lending, tuition vouchers, subsidized fruits and vegetables, massive increases in supervised physical activity, and inner-city health clinic expansion.

Perhaps we should tailor our approach to the reality that population health is ultimately local, a function of community well-being and ingenuity. If communities are the mechanisms of action, we may need to let them figure it out for themselves, supported by community-level incentives. The California Endowment (13) has funded 14 communities to pursue goals such as reduced childhood obesity, increased school attendance, reduced youth violence, and a "health home" for all. Suppose the president or Congress offered municipalities large prizes for achieving concrete health gains in a decade — say, a check for $100 million for a community of 100,000, or $1,000 per capita, payable on January 1, 2022 (baseline data would be gathered in 2011, and the clock would start ticking 1 year later). Methodologic issues would have to be addressed, but these are not insurmountable. Such incentives might galvanize coalitions of leaders, business people, educators, and community groups to take population health seriously. If the whole country got the maximum bonus, the federal government would pay $300 billion (300 million people × $1,000), or $30 billion per year. That's barely the rounding error on the size of the 2009 US economic stimulus package, and the very structure of the investment would guarantee an excellent return on investment in terms of both health and productivity.
